# Gender difference towards information and communication technology awareness in Indian universities

**DOI:** 10.1186/s40064-016-2003-1

**Published:** 2016-03-24

**Authors:** Chaman Verma, Sanjay Dahiya

**Affiliations:** Department of CSE, JJT University, Jhunjunu, Rajasthan India; Department of CSE, Ch. Devi Lal State Institute of Engineering and Technology, Panniwala Mota, Sirsa, Haryana India

**Keywords:** Hypothesis, Mean, Standard deviation, T test, Likert-scale

## Abstract

Nowadays, information and communication technology is major backbone of Indian education system. To support E-learning in Universities, information and communication technology (ICT) plays a momentous job. Several experts discussed about ICT awareness among students, teachers, and research scholars to take it into their learning and teaching methodology. Many of Universities either government or private are supporting the utilization of various ICT tools in teaching and learning practice. There is wide need to determine educator’s behaviour towards ICT adoption to promote and enhance their learning skills. Students and faculty must confess that ICT awareness is key rod to access the technological services. This paper focuses on ICT awareness among students and faculty residing in Indian Universities. The concerned paper is describing the attitude of students and faculty towards ICT awareness in relation to their gender using statistical tools. More than nine hundred samples have been gathered from six Indian universities. The findings of this paper will help to Indian Universities administration to get aware about current scenario of ICT involvement in education system therein.

## Background

The development of any country depends upon the well succeed education system. In India higher education plays a significant role in order to develop our youth. Nowadays, the technology is rapidly growing over the globe. ICT has covered up almost educational institutions in developed countries. Many countries have admitted in adopting ICT in their educational system as key of success. A landmark initiative in this regard Ministry of human resource and development India launched “The National Mission on Education through Information and Communication Technology”. Under this Mission, a proper balance between content generations, research in critical areas relating to imparting of education and connectivity for integrating our knowledge with the advancements in other countries is to be attempted. For this, what is needed is a critical mass of experts in every field working in a networked manner with dedication (Alten Group 2015). In India nearly 400 universities have been provided 1 Gbps connectivity or have been configured under the scheme and more than 14,000 colleges have also been provided Virtual Private Network connectivity (Beena Mathur 2012). The Government of India has taken ICT initiatives in a big way and has laid down a National ICT policy, which is reflected and implemented through various Government Departments and Ministries. It is being implemented through energetic activities of National Informatics Center and encouragements form University Grants commission, all India council of Technical Education and Department of Science and Technology throughout the country (Sharma and Singh 2010). After large implementation by Indian government it is extensive need to investigate the opinions of both students and faculty those are the key element in success of this project. Many of researchers have investigated the attitude of students and faculty regarding ICT awareness. Kozma (2003) stated that ICT allows teaching and learning activities by educational innovations and by connecting students and teachers to each other and to a vast array of human and informational resources around the world. Philomina and Amutha (2016) concluded those science teachers are more aware about ICT use in teaching as compare to arts teachers. Similarly female teachers won from male teachers in ICT awareness. Beena Mathur (2012) found that male students have shown higher awareness as compare to female students for the use of ICT in education. There is no significance difference between opinions of male and female students. Thakur (2014) revealed that there is no significant difference in the level of ICT awareness among the male and female trained teachers (Philomina and Amutha 2016). Pratik (2013) concluded that male and female B.Ed. students have similar attitude towards computer. There is no significance difference towards computer in relation to their student’s gender. Illayaperumal (2010) found that there is significant difference observed between the groups regarding locality, type of selection and community. Therefore, it is necessary for our future teachers to have the knowledge and understanding of the role of ICT in sustainable development. Dubey (2010) concluded that female faculty have more positive attitude towards computer as compare to male teachers.

## Objectives and hypothesis

To study the significant gender wise difference, test questionnaire among students and faculty towards information and communication technology awareness in terms of its availability/strength, usability/opportunity, problems/weaknesses/threats and solution of problems/weaknesses/threats, the objectives with their respective hypotheses have been designed:

1. To study opinion of students towards the information and communication technology awareness in relation to their gender.
2. To study opinion of faculty towards the information and communication technology awareness in relation to their gender.
3. To study opinion of overall male and female towards the information and communication technology awareness in relation to their gender.

To accomplish the above mentioned objectives null and their corresponding alternate hypothesis are described below:

### **H01**

There is no significant difference between boys and girls student’s opinions towards information and communication technology awareness.

### **HA1**

There is a significant difference between boys and girls student’s opinions towards information and communication technology awareness.

### **H02**

There is no significant difference between male and female faculty’s opinions towards information and communication technology awareness.

### **HA2**

There is a significant difference between male and female faculty’s opinions towards information and communication technology awareness.

### **H03**

There is no significant difference between overall male and female’s opinions towards information and communication technology awareness.

### **HA3**

There is a significant difference between overall male and female’s opinions towards information and communication technology awareness.

## Design and methodology

In present paper, quantitative method has been used. A normative survey technique has been conducted to gather primary data and to confirm the assumed null hypotheses. Students and faculty were asked to filled-up the questionnaire with objective to gather their opinions towards ICT awareness. The research design includes the following.

### Instrument design and validation

A structured questionnaire has been designed to gather opinions according to objectives. To gather samples of students and faculty from various universities, five point Likert format has been used. This instrument consisted of 35-items self-report scored on a 5 point Likert type scale [strongly disagree (SD) = 1, disagree (D) = 2, undecided (UD) = 3, agree (A) = 4, and strongly agree (SA) = 5]. The instrument or questionnaire has been divided into two major sections, which contained 35 dependent variables.

Table 1 displays two sections of questionnaire. Section first contains 10 and 08 dependant’s variables in relation to availability and usability of ICT awareness respectively. Section second contains 03, 05 and 09 dependant’s variables in relation to problems, solutions and opportunities of ICT awareness respectively. Face validity and content validity of the scale was ensured through consultation with faculty members from senior faculty members of participated universities. Item analysis test (DV–DP test by Kelley’s in 1939) has been applied to selection of variables. The present paper includes the 2 independent and 35 dependants’ variables. Gender is considered as independent variable and 35 dependents variables are chosen in accord with ICT awareness (availability, usability, issues and solutions) to investigate the opinions of students and faculty both students and faculty.

**Table 1:** Instrument dependant variables

*Section-I*	
Availability	
1	Adequate ICT infrastructure is available
2	Institution campus is Wi-Fi
3	Sufficient bandwidth is available for Internet
4	ICT tools/software are easy accessible
5	Institutions have clear policy framework to integrate ICT
6	Sufficient funds are available to promote ICT based research and development
7	Sufficient ICT tools/software and hardware are available in research laboratory
8	Institutions have E-library
9	Adequate E-journals/E-contents are available in library
10	E-contents are easily accessible/subscribed in library
Usability	
11	ICT used in Planning and Management
12	ICT tools/software used in research and development are reliable
13	Use of ICT encourages research and project development
14	ICT is used to exchange the research information with other organizations
15	ICT used adequately in teaching, learning and research activities
16	E-journals/E-contents effectively using in research and development
17	ICT is used to access the E-contents from other libraries
18	ICT is used to learn the lecture/lesson from other institutions experts through video conferencing
*Section-II*	
Problems	
19	Time consuming to integrate ICT into teaching, learning, research and development
20	Lack of readiness to adopt ICT technology in teaching and learning
21	ICT tools/software not user friendly due to lack of training
Solutions	
22	Need to increase the latest ICT infrastructure
23	Internet bandwidth should be increased
24	Need to increase E-journals/E-contents as per requirement
25	Need to enhance ICT in teaching and learning
26	Need for training/workshop to learn ICT tools/software and equipment
Opportunities	
27	ICT increase the effective teaching and E-learning in classroom
28	Students and Teachers feel more professional, motivate, confident while using ICT resources
29	ICT provides more comprehensive material of a particular topic
30	ICT plays an important role in admission and examination
31	Integrate of ICT increase placement activities
32	ICT reduce the cost for information exchange
33	ICT helps in design to new projects in higher education
34	Successful ICT integration will brighten the future of higher education
35	Using the ICT available increases productivity in higher education

Table 2 shows that 35 variables were found failed to reject out of 70 by achievement test and 2 variables (dv value >0.75) and 33 variables (DV is <0.20) were failed to accept.

**Table 2:** Difficulty value of dependant variables

S. no.	DV	Frequency	Variable no.	Remarks
1	>0.75	02	23, 45	Failed to reject
2	0.20–0.75	35	7, 8, 9, 12, 14, 16, 18, 19, 24, 25, 28, 31, 32, 33, 36, 37, 41, 43, 44, 46, 47, 48, 49, 50, 51, 52, 54, 55, 56, 58, 59, 61, 62, 64, 66	Failed to reject
3	<0.20	33	1, 2, 3, 4, 5, 6, 10, 11, 13, 15, 17, 20, 21, 22, 26, 27, 29, 30, 34, 35, 38, 39, 40, 42, 53, 57, 60, 63, 65, 67, 68, 69, 70	Failed to accept

Table 3 shows that 26 items were considered very good and no need for alteration (DP is range of 0.40–0.9) and 09 variables were considered good (DP is range of 0.30–0.39) needs little bit alteration; 35 items were found fail to accept due to poor status (<0.19). The present paper contains six dependant variables like boy, girl, male, female and overall male and overall female as per objectives.

**Table 3:** Discriminating power of variables

S. no.	DP	Frequency	Variable nos.	Remarks
1	0.40–0.9	26	9, 12, 14, 16, 18, 19, 23, 25, 31, 32, 33, 36, 37, 41, 43, 46, 47, 48, 49, 50, 51, 52, 54, 55, 56, 61	Very good
2	0.30–0.39	09	7, 8, 24, 28, 30, 44, 59, 62, 66	Good
3	<0.19	35	1, 2, 3, 4, 5, 6, 10, 11, 13, 15, 17, 20, 21, 22, 26, 27, 29, 30, 34, 35, 38, 39, 40, 42, 53, 57, 60, 63, 65, 67, 68, 69, 70	Poor

### Population identification

Students and faculty have been involved from various reputed universities in India. Both students and faculty members from various private and Government Universities have participated willingly. They are providing or receiving higher education in different fields like engineering, humanities and science field. Demographic characteristics of participants are given in Table 4.

**Table 4:** Participated universities

UNI’S	Haryana			Punjab			Total
	Govt.		Private	Govt.	Private		
	CDLU	GJUST	SGT	PU	CU	GKU	
N	144	138	148	143	198	133	904
%	15.9	15.3	16.4	15.8	21.9	14.7	100

Table 4 shows that 904 students and faculty from various universities located in Punjab and Haryana state of India, have participated in the present study. There are three Universities from each state. The name and affiliation status of Universities are given below:

1. Ch. Devi Lal University (CDLU)—Government.
2. Guru Jambheshwar University of Science and Technology (GJUST)—Government.
3. Shree Guru Gobind Singh TriCentenary University (SGT)—Private.
4. Punjabi University (PU)—Government.
5. Chandigarh University (CU)—Private.
6. Gurukashi University (GKU)—Private.

### Sampling

In present study, the stratified random sampling method is used. Nine hundred four students and faculty have been participated in this research from various Universities located in Punjab and Haryana state of India.

Table 5 shows that out of total 560 students, 274 (48.9 %) boys and 286 (51.1 %) girls have participated. Similarly out of total 344 faculty 175 (50.9 %) male faculty and 169 (49.1 %) female faculty have participated. All students and faculty belong either to graduate level or post graduate level.

**Table 5:** Gender distribution of participants

S. no.	Particular	Students #n = 560		Faculty #n = 344		Total #n = 904	
		Boys	Girls	Male	Female	Male	Female
1	Frequency (n)	274	286	175	169	449	455
2	Percentage (p)	48.9	51.1	50.9	49.1	100	

### Statistical techniques

To analyse the data collected from various Universities standard statistical techniques are used. To test the proposed hypotheses to achieve objectives, student T test has been applied. Beside of this descriptive analysis [frequency (N), percentage (%), mean and standard deviation] have been also implied in present study. To determine significant difference among students and faculty opinion in relation to their gender T test with equal variance has been applied using Microsoft Excel with extra Add-ins named Analysis Toolpack and Analysis Toolpack-VBA.

## Findings

In this section results have been found regarding analysis of students and faculty opinions in relation to their gender. The results of the independent group’s T test have been applied to test assumed hypothesis.

## Testing of hypothesis H01

It is evident from Table 6 that the calculated T value is 0.61, is smaller than the critical table value which is 2.0 with degree of freedom 68 at 5 % level of significant (0.61 < 2.0 with df = 68 at 0.05 significant level). Hence it is not significant up to 5 % level. Therefore, it is reflecting that gender variable did not influenced student’s opinions towards ICT awareness.

**Table 6:** Gender wise student’s opinions analysis using T test

	Boys	Girls
Mean	3.58	3.64
Standard deviation	0.39	0.38
Variance	0.151	0.145
T value	0.61 at df = 68 T critical two-tail = 2.0	

Hence, first null hypothesis H01 “There is no significant difference between boys and girls student’s opinions towards information and communication technology awareness.” is failed to reject here and another side alternate hypothesis HA1 “There is a significant difference between boys and girls student’s opinions towards information and communication technology awareness.” is failed to accept here. Therefore, there is no significant difference found between boys and girls student’s opinions towards information and communication technology awareness.

**Fig. 1:**
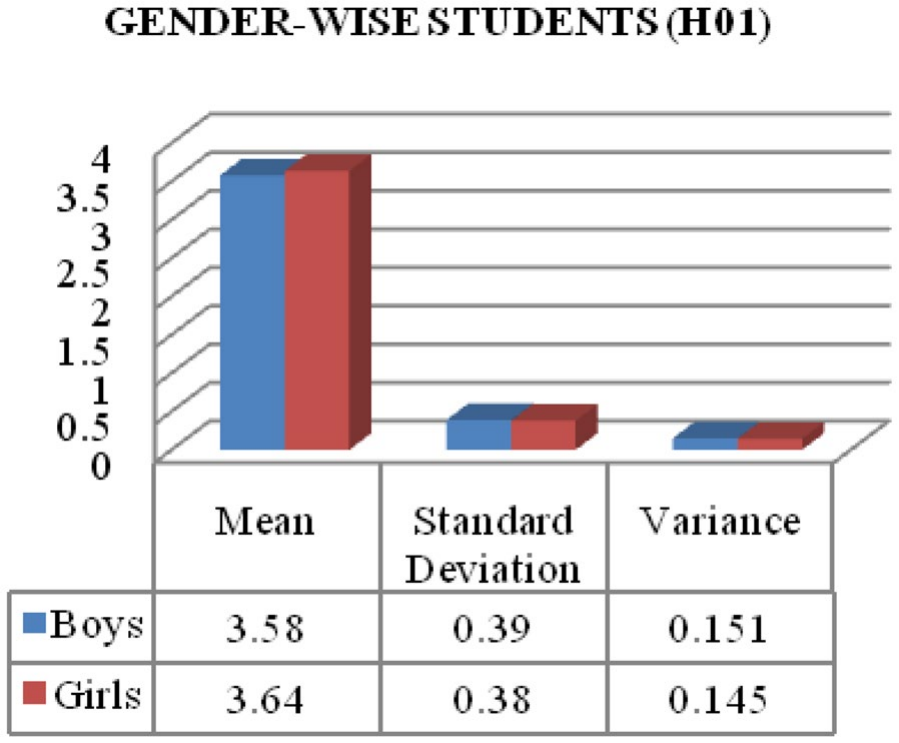
Gender wise opinions difference among students

Above Fig. 1 it is clearly showing that boys and girls students have not significant difference in their opinions towards ICT awareness. Boys and girls students mean values are found 3.58 and 3.64, respectively. Standard deviations and variances for responses of boys and girls are also reflecting no meaningful difference between them. Findings of this test support the results of Thakur (2014). The mean score 3.58 of boys are deviated from standard deviation 0.39, which shows their range of responses lies in between 3.19 and 3.97. This reflects boys student are agreed to accept awareness about ICT. Similarly girl’s mean value 3.64 is deviated from standard deviation 0.38, which shows their range of replies lie in between 3.26 and 4.02. Hence, girls were found little bit more aware about ICT.

## Testing of hypothesis H02

Data from Table 7 is concluded that calculated T value is 0.58, is smaller than the critical table value, which is 2.0 with degree of freedom 68 at 5 % level of significant (0.58 < 2.0 with df = 68 at 0.05 significant level). Hence it is not significant up to 5 % level. Therefore, it is found that gender variable did not affect faculty’s opinions towards ICT awareness.

**Table 7:** Gender wise faculty opinions analysis using T test

	Male	Female
Mean	3.83	3.86
Standard deviation	0.25	0.21
Variance	0.06	0.04
T value	0.58 at df = 68 T critical two-tail = 2.0	

Hence, second null hypothesis H02 “There is no significant difference between male and female faculty’s opinions towards information and communication technology awareness.” is failed to reject here and another side alternate hypothesis HA2 “There is a significant difference between male and female faculty’s opinions towards information and communication technology awareness.” is failed to accept here. Therefore, there is no significant difference found between male and female faculty‘s opinions towards information and communication technology awareness. Findings of this paper support the results of Pratik (2013).

**Fig. 2:**
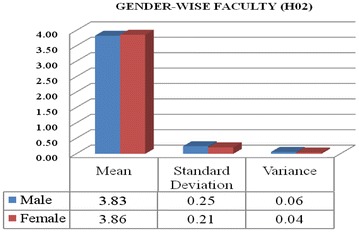
Gender wise opinions difference among faculty

Figure 2 it is showing that male and female faculty have not significant difference in their opinions towards ICT awareness. The male and female faculty’s mean values are found 3.83 and 3.86, respectively. Standard deviations and variances for both are also reflecting no meaningful variation between them. Both male and female faculty have admitted that they are aware regarding ICT awareness. The mean value for female is 3.86 is deviating from standard deviation 0.21, which shows that responses lies in between the range of 3.65–4.07 (undecided to strongly agree).

## Testing of hypothesis H03

Data from Table 8 is concluded that calculated T value is 0.57, is less than the critical table value which is 2.0 with degree of freedom 68 at 5 % level of significant (0.57 < 2.0 with df = 68 at 0.05 significant level). Hence, it is not significant up to 5 % level. Therefore, it is found that overall male and overall female have not meaningful difference towards ICT awareness.

**Table 8:** Overall opinions analysis using T test

	Over all males	Over all females
Mean	3.68	3.72
Standard deviation	0.32	0.30
Variance	0.10	0.09
T value	0.57 at df = 68 T critical two-tail = 2.0	

Hence, third null hypothesis H03 “There is no significant difference between overall male and female’s opinions towards information and communication technology awareness.” is also failed to reject here and another side alternate hypothesis HA3 “There is a significant difference between boys and girls student’s opinions towards information and communication technology awareness.” is failed to accept here. Therefore, there is no significant difference found between overall male and female’s opinions towards information and communication technology awareness. Hence, outcomes of this study are opposing with the findings of the researcher Philomina and Amutha (2016).

**Fig. 3:**
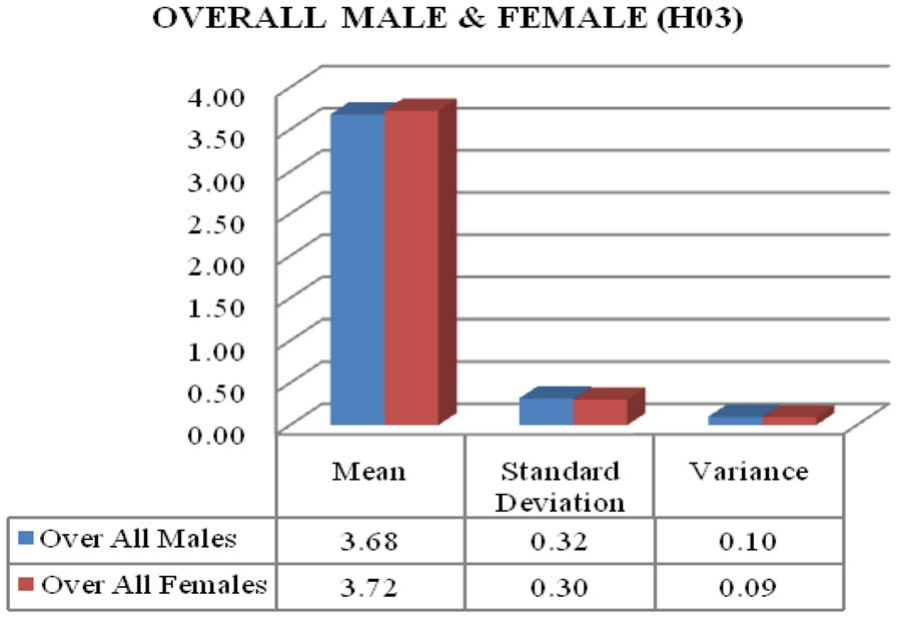
Overall opinions difference

Figure 3 is also confirming that overall males (boys student and male faculty) and females (girl’s student and female faculty) have same opinions towards ICT awareness. Mean score of overall males are 3.68 and overall females are 3.72. The overall female’s mean scores is looking little bit more as compare to overall male but not seems as significant. The mean value for overall females is 3.72 is deviating from standard deviation 0.3, which shows that responses lies in between the range of 3.42–4.02 (undecided to strongly agree).

## Conclusion

This study has been carried out to test the statistically significantly difference in students and faculty opinions towards ICT awareness in relation to their gender variable. This study was conducted on educators and students of various Universities locating in Haryana and Punjab state of India. It has been concluded that gender variable did not affect students and faculty opinions towards information and communication awareness. It has revealed that there is no statistically significant difference between boys and girls students towards ICT. The findings of this paper are also proving that there is no meaningful difference between male and female faculty towards ICT awareness. The results of study have provided suggestions that there is ardent need for availability of ICT infrastructure in Universities campuses. The present research is carried out in order to aware real scenario of ICT awareness in terms of usability, availability, problems and opportunities/solution, which have never been analyzed by mentioned literature. This research is significant to postgraduate, graduate, research scholars, and educators in lengthening their perceptive and awareness about real aspects, which effects ICT adoption, usability and availability, crises and solutions of crises facing by them especially in educational sector. This study is also beneficial and academically important for them because there has been little experimental based research emphasizing on the numerous factors of faculty awareness of ICT implementation in Higher education and research. It is the prime discovery to believe responses made Indian faculty and students relating to ICT awareness in higher education. It is also concluded that there is high involvement of information and communication technology awareness in Indian higher education and research institutions. The findings of present study provide suggestions to both states administrations and Universities’ authorities to provide more ICT resource facility in academic institutions to promote technological based quality education.
